# sortscore: Sort-seq MAVE scoring and visualization using Python

**DOI:** 10.1093/bioinformatics/btag664

**Published:** 2026-09-02

**Authors:** Caitlyn Chitwood, Emily Orr, Dustin Baldridge

**Affiliations:** Department of Pediatrics, Washington University School of Medicine, St. Louis, MO 63110, United States; Department of Pediatrics, Washington University School of Medicine, St. Louis, MO 63110, United States; Department of Pediatrics, Washington University School of Medicine, St. Louis, MO 63110, United States

## Abstract

**Summary:**

A growing number of tools enable the analysis of large variant libraries produced by multiplexed assays of variant effects (MAVEs). Experiments using fluorescent reporters and fluorescence-activated cell sorting sequencing (FACS-seq or Sort-seq) can coarsely quantify a given variant’s impact on phenotypes such as transcription activity. Existing bioinformatics tools for Sort-seq data broadly fall into two categories: methods that model a genotype-phenotype landscape to infer latent variant phenotypes, and methods that directly estimate individual variant scores from experimental binned counts. Within this second category, sortscore provides an activity score computed directly from observed counts without fitting a model, retaining the original experimental scale when bin median values are known. We present sortscore, a python package that incorporates a standard Sort-seq scoring method and normalization across sorted samples, technical replicates from separate sort times, and across oligos in tiled experiments. It also provides convenient heatmap visualizations. This software was used to analyze DMS experiments for the transcription factor GLI2. This work seeks to lower the barrier to entry and provide a clear starting point for scoring Sort-seq cell-based functional assays. This extends the availability of well-documented and reproducible data analysis protocols to a wider community employing MAVE techniques.

**Availability and implementation:**

The package can be downloaded from PyPI using the pip installer. The code is also freely accessible and available for reuse through a Public GitHub repository (MIT License). Installation instructions, documentation, and tutorials are accessible at the sortscore GitHub repository: https://github.com/dbaldridge-lab/sortscore. A snapshot of the code and data is available on Zenodo: https://doi.org/10.5281/zenodo.22119031.

## 1 Introduction

### 1.1 MAVE Background

Multiplexed assays of variant effects (MAVE) are cell-based assays used to measure experimental readouts of biological function and estimate the impact of thousands of genetic variants in a single experiment (Kinney 2010). MAVE methods include deep mutational scanning (DMS), in which amino acid residues are systematically replaced via saturation mutagenesis to comprehensively evaluate single-nucleotide variants (Tabet *et al*. 2022), multi-nucleotide edits within individual codons, or combinations of edits across residues to measure epistatic effects. The application of MAVE methods has grown rapidly since their introduction, in part due to their demonstrated ability to elucidate biological mechanisms through genetic perturbations (Fowler and Fields 2014, Esposito *et al*. 2019) and attribute clinical outcomes to specific variants in a disease gene (Findlay *et al*. 2018, McEwen *et al*. 2026). Functional datasets also serve as promising gold-standards in the validation of computational variant effect predictors (Livesey and Marsh 2020).

Growth phenotypes are the most common DMS experimental outcome, and most MAVE software developed to date has been designed to accommodate growth assays (Çubuk *et al*. 2025). However, fluorescent reporters in combination with fluorescence-activated cell sorting (FACS) and subsequent DNA sequencing–referred to here as Sort-seq–are also employed in the MAVE literature (Matreyek *et al*. 2018, Ma *et al*. 2024, Aslanzadeh *et al*. 2025, Popp *et al*. 2025), including in massively parallel reporter assays (MPRAs) (Peterman and Levine 2016).

### 1.2 Sort-seq background

Sort-seq involves sorting a population of input cells into tubes defined by gating on a phenotype measured at single cell resolution, most commonly fluorescence intensity level, followed by sequencing the DNA from those sorted cells. An overview of the Sort-seq workflow is shown in Supplementary Fig. 1, available as supplementary data at *Bioinformatics* online. This approach can be advantageous when a clinical phenotype or gene function may not be well correlated with cell proliferation and survival, and when fluorescent reporters are available to assess cellular function, such as reporters responsive to transcription factor activity (Shepherdson *et al*. 2024). Beyond functional testing of genetic variants in the context of human health, Sort-seq methods have also been used to monitor bacterial populations (Lambrecht *et al*. 2017) and to estimate variant impacts on protein properties such as stability and solubility (Gaber *et al*. 2024). Sort-seq generally requires the incorporation of a fluorescent reporter into the biological pathway being studied, which may require cell line engineering.

The Sort-seq experimental process produces a count distribution for each population of cells carrying any one of potentially thousands of variants. Cells are sorted into discrete bins, each spanning a select interval out of the full dynamic range of the perturbed fluorescence phenotype (shown as bins in Supplementary Fig. 1.4, available as supplementary data at *Bioinformatics* online). Resulting sequencing files are processed to map sequencing reads back to mutagenesis variants. The sequencing read counts from each sorted sample reflect the relative abundance of each variant. These processed read counts become inputs for downstream analyses, such as those performed in our Python package, sortscore.

### 1.3 Sort-seq scoring software

Compared to growth assays, analytical software support for scoring Sort-seq MAVEs remains limited but is improving. The Lilace R package began filling this gap by providing an implementation of a Bayesian hierarchical scoring framework to estimate latent unimodal shifts in the fluorescence distribution for variants relative to a negative control baseline (e.g. synonymous distribution) (Freudenberg *et al*. 2026).

We developed sortscore, which implements a weighted mean scoring approach to provide an additional, dedicated, reproducible tool for Sort-seq analysis. It incorporates experimentally derived bin weights–in the case of FACS, median fluorescence intensity (MFI)–when available, to retain quantitative detail and experimental units that are discarded when bins are treated as ordinal categories. Since MFI values are not strictly required, this approach can readily generalize to other bin-based MAVE assay designs beyond FACS-based Sort-seq. In its current implementation, sortscore processes sequencing read count data generated by FACS-based assays and provides activity scoring through a streamlined CLI workflow, along with visualization options (64 codon heatmaps and tiled heatmaps) and tiled normalization for short-read sequencing contexts–features which are not currently implemented in other Sort-seq MAVE tools.

While sortscore and Lilace directly score individual variants via weighted mean and Bayesian hierarchical methods respectively, a branch of related tools models global genotype-phenotype landscapes from which variant-level phenotypes are then inferred. MAVE-NN is one such tool which accepts binned counts to train a neural network that deconvolutes measurement noise and nonlinearities (Tareen *et al*. 2022). LANTERN and MoCHI apply low-dimensional and mechanistic biophysical approaches, respectively (Tonner *et al*. 2022, Faure and Lehner 2024). The methodological differences between these tools are described further in Table 1 below.

**Table 1:** Comparison of selected MAVE analytical tools that provide variant-level activity scoring or phenotype prediction for Sort-seq experiments.

Software	Type	Summary	Variant Score / Phenotype Prediction	Uncertainty Quantification
Sortscore	Variant Scoring from binned counts	Quickly score variants and normalize tiled Sort-seq experiments	MFI bin weights produce a variant fluorescence estimate; ordinal bin weights produce a rank-based score	Not presently implemented
Lilace		Robust FDR control for DMS employing FACS	Latent unimodal shift in fluorescence distribution relative to negative control	Negative control-based bias correction; local false sign rate (lfsr)
MAVE-NN	Genotype-Phenotype Landscape Modeling	Learn quantitative G-P maps and deconvolute noise and nonlinearities	Output of the nonlinear function transforming the latent phenotype into measurement units	Parametric bootstrap approach
LANTERN		Find low-dimensional feature space for interpretable discovery	Posterior Mean of the nonlinear surface	Posterior predictive uncertainty
MoCHI		Fit mechanistic models to quantify absolute energies and interactions	Predicted observed phenotype that converts molecular phenotype into units of assay	Monte Carlo confidence intervals

Beyond these methodological distinctions, the customizable workflows implemented by sortscore lower barriers to Sort-seq analysis. Since weighted mean scoring has been commonly applied historically, sortscore facilitates reproduction of these prior analyses within a standardized framework. Its CLI implementation further provides an accessible option for researchers working in high-performance computing environments or incorporating scoring into a larger data pipeline.

## 2 Methods

### 2.1 Software design

#### 2.1.1 Standardized analysis workflows

Figure 1 shows two standardized analysis workflows that can be executed using the command-line interface. These console commands offer a convenient way to add scoring and visualization to the end of a sequencing data processing pipeline, as in compute environments with batch job schedulers. The Variant Scoring workflow takes processed sequencing counts files from Sort-seq experiments and returns a heatmap and a Score Set CSV file. Prior to normalization, batch effects can be apparent across Sort-seq experiments. The Batch Normalization workflow takes Score Set files from multiple tiled experiments, and returns a combined Score Set and heatmap on a unified scale for comparison.

**Figure 1:**
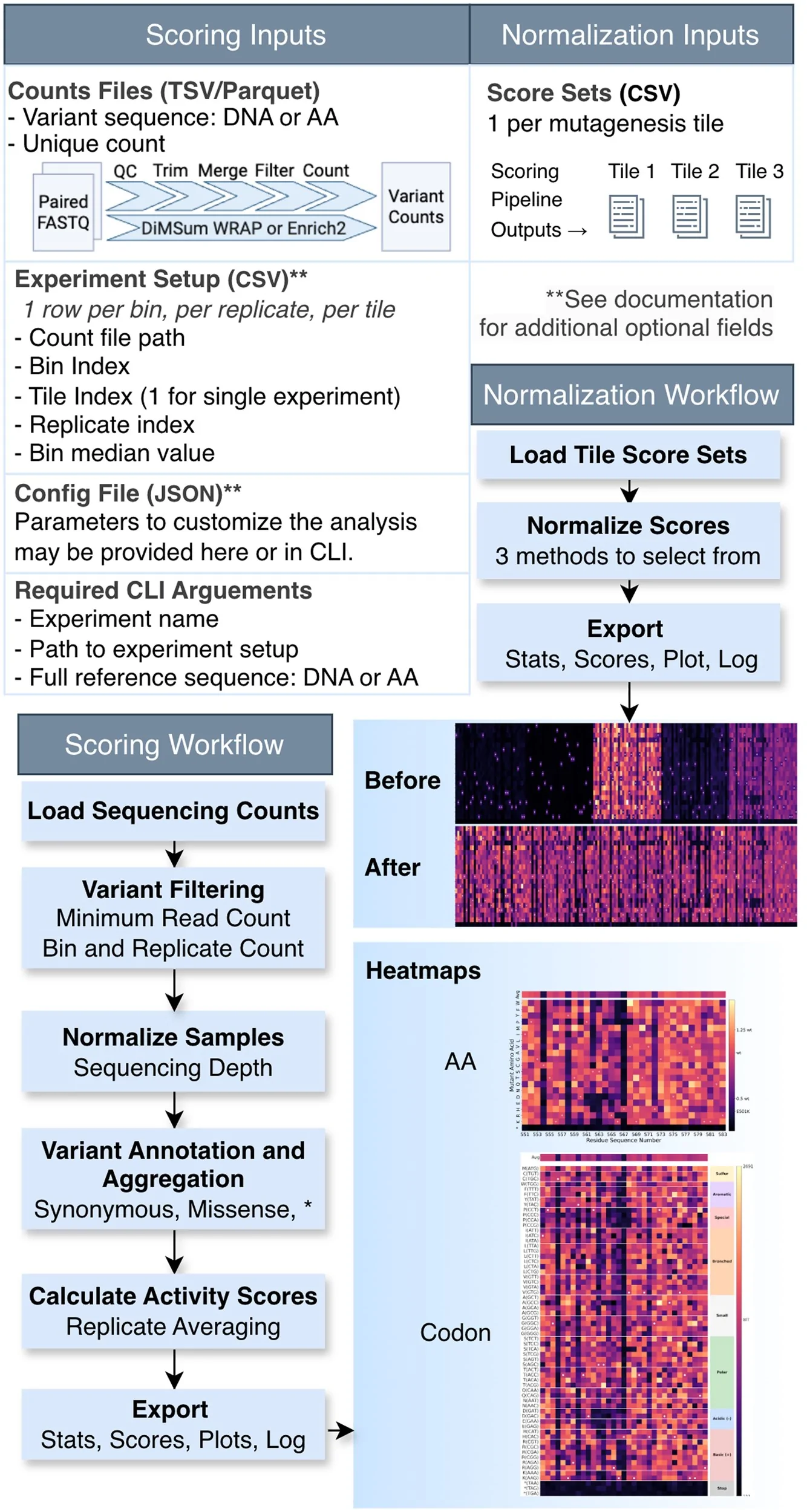
Command-line workflows for variant scoring and batch normalization. The Variant Scoring workflow processes Sort-seq count data through filtering, depth normalization, variant annotation, and score calculation to generate score sets and heatmaps. The Batch Normalization workflow combines score sets across tiled experiments onto a common scale. Alt text: Flowchart showing two command-line workflows: Variant Scoring converts sequencing counts into variant scores and heatmaps; Batch Normalization combines tiled Score Sets into normalized scores, plots, and logs.

#### 2.1.2 Dependencies

The sortscore package utilizes standard scientific Python libraries including NumPy and pandas for data processing, SciPy for statistical analysis, matplotlib and seaborn for visualization, and Biopython for biological sequence handling (Hunter 2007, Cock *et al*. 2009, Harris *et al*. 2020, Virtanen *et al*. 2020, Waskom 2021, The pandas development team 2026).

#### 2.1.3 Data loading and input requirements

The sortscore package is focused on calculating variant scores based on relative abundance in sorted bins, normalizing data across tiled sorting experiments, and visualizing these results via heatmaps. Sortscore accepts variant counts files in TSV (gzipped or uncompressed) and Parquet formats, requiring that they contain two columns: sequence (or sequence identity) and unique counts. Currently available software is routinely used to convert raw sequencing files into the input variant counts file. Such tools include Enrich2 or DiMSum’s WRAP module for short reads, or Pacybara for long reads (Rubin *et al*. 2017, Faure *et al*. 2020, Weile *et al*. 2024). Software for mapping barcodes to variants is available in the Call Variants DMS Docker container (P-Ruzycki/Call-Variants-DMS-Docker).

Variants with low read counts may be filtered during loading by specifying a threshold in units of reads per million in the configuration file. This minimum read filter applies at the bin level and is 0 by default.

### 2.2 Analysis configuration

#### 2.2.1 Configuring scoring for a single sorting experiment

Easily modifiable configuration files provide flexibility to tailor the analysis workflows to new experimental assays using a basic text editor. Configuration for running sortscore is provided via two files: (a) a JSON configuration file, and (b) a CSV file to define the experimental design. Templates for each are available on GitHub: config.json and experimental_setup.csv, respectively. The project documentation on GitHub also lists the full set of required and optional fields available to customize the scoring workflow.

Modifiable fields in the JSON include the mutagenesis approach (e.g. all possible amino acids per position, presence or absence of nonsense variants), quality filter thresholds, and visualization customizations. The experiment setup CSV file provides the structure to share file paths, information about technical replicates, sum of read counts per sample, and gating strategy for a single sorting experiment.

#### 2.2.2 Configuring normalization across tiled experiments

The same configuration file can be used to select the desired normalization method, specify negative controls to use during normalization, and provide paths to the configuration files for individual experiments that will be combined. The three available methods for normalization are described below. Prior to normalization across tiles, count files for each tile must be processed using the Variant Scoring workflow.

#### 2.2.3 DNA and AA variant scoring and heatmaps

Since a wide range of mutagenesis approaches are used in MAVE experimental designs, we provide a limited set of variant aggregation and heatmap customization options. When DNA sequences or variant identifiers are provided, users can specify whether scores and heatmaps should reflect the DNA-level variant or protein sequences that have been aggregated across codons. The package auto-detects the type of sequences present in the counts files and will alert the user if this is incompatible with the requested scoring option.

### 2.3 Variant activity scoring

The primary function of the first sortscore analysis pipeline is to return an activity score for each binned variant. To quantify activity, we estimate the mean phenotypic value utilizing elements from the discrete counts distribution for each variant. In Sort-seq, these distributions are produced by sorting cells into tubes corresponding to intervals of the measured range of phenotype values followed by sequencing. This simple estimator is practical, easy to understand and compute, and is useful for ranking variants and comparing resulting scores against the activity of benign-like and/or pathogenic-like control variants.

#### 2.3.1 Normalization across samples

The package controls for sequencing depth differences by normalizing total read counts across samples as reads per million (RPM). Total reads in a sample can be user-provided or calculated from the counts files.


$${\text{RPM}}_{v,b}=(\frac{\text{read}\;{\text{count}}_{v,b}}{\text{total}\;{\text{reads}}_{b}})\cdot 10^{6}$$

v=Variant indexb=Bin index

#### 2.3.2 Score calculation

For each variant, Sort-seq produces a discrete distribution defined by ordered fluorescence bins and the counts of variant sequencing reads observed in each bin. This variant fluorescence distribution can be modeled as a probability mass function. FACS discretizes the underlying continuous fluorescence distribution of the cell population containing a given variant (see Supplementary Fig. 2, available as supplementary data at *Bioinformatics* online). The sortscore package calculates an expected fluorescence score, Sv, as a weighted mean across bins, weighting the proportion of variant reads in each bin by a user-provided bin weight and summing across bins. This weight is typically the bin MFI for experiments employing FACS, but any user-defined value can be substituted, including an integer-based ordinal value. MFI-based weights capture the actual, and often unevenly spaced, differences in intensity between bins, whereas ordinal weights assume evenly spaced ranks. Despite this simplification, we found ordinal-weighted scores to be highly concordant with MFI-weighted scores in a demo dataset with unevenly spaced bins (see Supplementary Fig. 3, available as supplementary data at *Bioinformatics* online). MFI-based bin weights produce an estimate of the variant-carrying cell population’s mean fluorescence. Ordinal bin weights produce a comparable rank-based score directly from binned counts, without fluorescence units. Both options serve as proxies for variant function.


$$S_{v}=\sum p_{v,b}w_{b}$$

$$p_{v,b}=\mathrm{proportion}\;\mathrm{of}\;\mathrm{reads}\;\mathrm{for}\;\mathrm{variant}\;v\;\mathrm{in}\;\mathrm{bin}\;b\;\mathrm{after}\;\mathrm{normalization}\;\mathrm{for}\;\mathrm{sequencing}\;\mathrm{depth}w_{b}=\mathrm{median}\;\mathrm{fluorescent}\;\mathrm{intensity}\;\mathrm{or}\;\mathrm{ordinal}\;\mathrm{value}\;\mathrm{assigned}\;\mathrm{to}\;\mathrm{bin}\;b$$

#### 2.3.3 Technical replicate averaging

By default, when averaging scores across technical replicates (defined as the same starting cell population sorted into bins in more than one FACS session), variant read counts serve as weights for calculating the mean across replicates. We use this weighting because we assume that higher read counts in a given replicate will result in more accurate scores for that replicate. The user can opt for a simple average instead in the analysis configuration.


$${\bar{S}}_{v,\text{weighted}}=\frac{\sum_{r=1}^{R}S_{v,r}\cdot w_{v,r}}{\sum_{r=1}^{R}w_{v,r}}$$

$$S_{v,r}=\mathrm{Activity}\;\mathrm{score}\;\mathrm{for}\;\mathrm{variant}\;v\;\mathrm{in}\;\mathrm{replicate}\;rw_{v,r}=\mathrm{Total}\;\mathrm{normalized}\;\mathrm{read}\;\mathrm{count}\;\mathrm{for}\;\mathrm{variant}\;v\;\mathrm{in}\;\mathrm{replicate}\;rR=\mathrm{Number}\;\mathrm{of}\;\mathrm{replicates}$$

#### 2.3.4 Normalization and scaling across tiled experimental batches

Our package provides two options for reducing potential batch effects that may be observed across different tiled experiments, by which we mean the use of separate oligos that code for different portions of the same protein. These adjustments to the activity scores bring them onto a similar scale for comparison.

#### 2.3.5 Method 1: linear range normalization

We provide this normalization method used in the 2018 publication of the BRCA1 saturation genome editing DMS (Findlay *et al*. 2018). An interval scale is set to the score difference between global nonsense and global synonymous score to bring batches into alignment and aid interpretability.


$$S_{\text{norm}}=b(\frac{A-C}{a-c})$$

$$b=\mathrm{Variant}\;\mathrm{activity}\;\mathrm{score}(\frac{A-C}{a-c})=\mathrm{Scaling}\;\mathrm{factor}\;(\mathrm{global}\;\mathrm{range}/\mathrm{local}\;\mathrm{range})a=\mathrm{Synonymous}\;\mathrm{median}\;(\mathrm{local})c=\mathrm{Nonsense}\;\mathrm{median}\;(\mathrm{local})A=\mathrm{Synonymous}\;\mathrm{median}\;(\mathrm{global})C=\mathrm{Nonsense}\;\mathrm{median}\;(\mathrm{global})$$

#### 2.3.6 Method 2: biological two-control anchoring, “two-pole” (default)

This normalization method (see Supplementary Fig. 4, available as supplementary data at *Bioinformatics* online) is the default used by sortscore and assumes that the tile-specific synonymous variant scores and nonsense variant scores should be similar to one another across experiments. It involves a three-step process: (1) normalization by the ratio of the global median ($M_{syn}^{global}$) and the tile-specific median ($M_{syn}^{tile}$) of synonymous variants, which all code for the same wildtype protein, (2) standardization of scores based on the scale of the distribution of synonymous scores, and (3) normalization by the ratio of the global and tile-specific medians of nonsense variants. The units of scores resulting from this method do not represent either the relative fold change in activity or the unit variance from a particular distribution, but rather should be interpreted in reference to positive and negative controls, when possible. However, this method does cause the directionality of activity change to be immediately apparent, because the mean synonymous score is centered at zero.


$$S_{v}^{\left(1\right)}=S_{v}\cdot \frac{M_{\mathrm{syn}}^{\mathrm{global}}}{M_{\mathrm{syn}}^{\mathrm{tile}}}$$

$$S_{v}^{\left(2\right)}=\frac{S_{v}^{\left(1\right)}-{\bar{S}}_{\mathrm{syn}}^{\left(1\right)}}{\sigma_{S_{\mathrm{syn}}^{\left(1\right)}}}$$

$$S_{\mathrm{norm},v}=\;S_{v}^{\left(2\right)}\cdot \frac{M_{\mathrm{nonsense}}^{\mathrm{global}}}{M_{\mathrm{nonsense}}^{\mathrm{tile}}}$$

#### 2.3.7 Method 3: biological single control anchoring, “one-pole”

This method is equivalent to Method 2, except that it omits the nonsense normalization factor to accommodate experiments that may not include multiple nonsense variants in each experimental batch. Instead, only the synonymous scaling factor is applied to scores by multiplying by the ratio of the global synonymous median and the tile-specific synonymous median.

#### 2.3.8 Quality control filtering

In an effort to improve the quality of the resulting scores, we provide options for filtering out or dropping values that may not meet certain thresholds. For example, a minimum replicates filter allows users to specify the number of technical replicates that each variant should be present in so that it can be included in the final score set of values averaged across replicates. The rationale for this filter is that if a variant is missing from multiple technical replicates, it may be poorly represented in the studied population, and any resulting scores may be unreliable due to insufficient sampling of that variant. A related minimum bins filter requires that a variant is represented in a given number of sorted samples in order to be assigned a replicate level score. This filter is highly context dependent because some experimental designs will intentionally yield zero counts in some bins, but as a guide one might consider the number of bins that control variants fall into. The default behavior is to not apply these filters.

### 2.4 Data and visualization outputs

#### 2.4.1 Plotting capabilities

A potentially particularly valuable feature to users is that sortscore produces codon and protein substitution heatmaps to visualize relative activity scores across variants. Position within the protein is oriented on the *y* axis and each variant is represented by a different row on the *x* axis. Optional biophysical property annotations may aid in interpretation of calculated scores. Positional average scores and coverage statistics are also included in the heatmap visualizations. Plotting functions can save outputs in PNG, PDF, or SVG formats.

#### 2.4.2 Data exports

Sortscore also returns full Score Sets, Score Set summary statistics, and the matrix of scores that are displayed in the heatmap. The heatmap matrix values are provided so the user can easily recreate and reformat the plot using other tools. This output will be raw scores if sortscore was run separately for individual experiments, or adjusted scores if run in batch mode. Summary statistics are also provided for different classes of variants in a JSON file, such as synonymous with wildtype versus nonsense versus all missense variants, allowing rapid assessment of how the scores were distributed in a given experiment.

### 2.5 MAVE ecosystem integration

MaveDB serves as a centralized repository for functional datasets and provides visualization functionality, with upcoming ClinVar submission integration (Esposito *et al*. 2019). Structured JSON configuration files and detailed output and error logging in sortscore enhance its interoperability with web applications, enabling potential integration with community resources such as MaveDB.

## 3 Results

### 3.1 Processing sort-seq data from GLI2 DMS

The data shown in the heatmap in Fig. 1 was produced in a series of DMS experiments performed by our lab with the aim of characterizing the pathogenicity of variants within the GLI2 transcription factor DNA binding domain, from a manuscript in preparation. Additional experimental details are provided in Supplementary Figs 1 and 2, available as supplementary data at *Bioinformatics* online.

A fluorescent reporter integrated into the genome of the NIH3T3 cell line produced GFP as a readout of intact transcriptional activity (Pusapati *et al*. 2018). GFP (FITC) fluorescence intensity was used to divide the cell population into four equal bins, each representing approximately 25% of the GFP expression distribution (see Supplementary Fig. 2, available as supplementary data at *Bioinformatics* online). DNA was then extracted and sequenced, and flow cytometry data were analyzed using FlowJo software.

Counts data were produced from FASTQ files using standard bioinformatics tools: cutadapt for trimming adapters (Martin 2011), VSEARCH for merging paired reads (Rognes *et al*. 2016), and a custom awk script used to confirm the read was a member of the list of designed sequences. As expected, the sortscore analysis prominently displays the deleterious impact of variants in the zinc finger domain as vertical dark stripes on the heatmap, particularly for the residues known to chelate the zinc ions (Pavletich and Pabo 1991).

The use of a sequential colormap for the heatmap improved interpretability of these GLI2 data compared to the standard red-blue versions commonly seen in MAVE publications which have great utility in the presence of both gain and loss of function missense variants. However, we have found no definitive evidence for gain of function behavior in the GLI2 DNA binding domain DMS to date. When only one prominent direction of effect is present, as in these data, sequential colormaps can make critical residues more apparent. The complete dataset will be made available upon publication of a manuscript currently in preparation, which also utilized sortscore for analysis.

We also tested the package by scoring an existing DMS dataset for the CRX gene using default options (Shepherdson *et al*. 2024). The resulting heatmap is shown in Supplementary Fig. 5, available as supplementary data at *Bioinformatics* online.

### 3.2 Tool comparison

Table 1 considers publicly available software packages that address the need for variant scoring in Sort-seq experiments. These separate broadly into two approaches, (1) genotype-phenotype landscape modeling methods that first model a surface for the region then predict latent phenotypes as a function of this feature space and (2) methods directly utilizing individual variant fluorescence distributions from experimental binned counts to produce estimates. The sortscore package adds to this ecosystem by providing an intuitive and direct activity score calculation in a user-friendly package. When the experimental bin median values are known, scores are returned in the original scale of the experiment that generated the data. Of the tools considered, Lilace is the closest comparator to sortscore in its goal of providing a dedicated tool for scoring FACS-based DMS data; we therefore compared sortscore estimates to those from Lilace on a FACS-based deep mutational scan of GLI2 residues 518–550, finding strong agreement between the two methods (Spearman’s *ρ* = 0.99, *n* = 693; Supplementary Fig. 6, available as supplementary data at *Bioinformatics* online). In Supplementary Table 1, available as supplementary data at *Bioinformatics* online, we summarize additional tools, primarily designed for growth assays, that are presently available for MAVE analysis and may be of interest to the reader.

## 4. Limitations and future development

### 4.1 Opportunities for improvement

Sortscore is focused on addressing scoring and visualization needs for Sort-seq experiments and avoids redundant implementation of functionality already provided by other, more established MAVE software. At present, users must generate input counts from raw sequencing data prior to running sortscore, though future integration work could streamline handoff from tools that perform this upstream step. This initial release implements an easy-to-interpret weighted average and basic quality filtering. Because this initial implementation computes scores directly rather than fitting a statistical model, uncertainty quantification is not built into the estimation method; as a result, sortscore outputs are best used as inputs for downstream MAVE interpretation rather than as a final analysis endpoint at this time. Additional next steps may include identifying variants with activity distinguishable from a reference background distribution, comparing score distributions across functional classes, integrating external annotations, and prioritizing variants for follow-up testing. Toward this end, sortscore already calculates a modified *z*-score during batch normalization, and this internal machinery could be extended in future work to provide a formal per-variant significance test, such as a *p*-value distinguishing variant activity from background.

Examples of analysis approaches that are adjacent to the sortscore package include the Oddspath calculation which can be used to determine an overall assessment of a functional assay’s ability to accurately discriminate benign from pathogenic variants (Brnich *et al*. 2018), and ExCALIBR, which can assign evidence levels to individual functional scores (Tejura *et al*. 2026). At a higher level, community-maintainable and reproducible approaches that bundle MAVE analysis tools might include containerizing complementary software via Docker or developing Nextflow modules that could be used with the deepmutscan nf-core pipeline (Wehnert and Stammnitz 2025).

### 4.2 Looking beyond FACS: applicability across cell sorting

Unexplored and underutilized sortable cellular phenotypes show potential for adding orthogonal layers of evidence to atlases of variant effects. Additional multiplexed measurement modalities compatible with sequencing would be valuable for directly quantifying variant effects and mapping their impact onto underlying biological mechanisms. Current sorting approaches coarsen phenotypic data, introducing significant noise at this stage of Sort-seq experiments. Throughput of millions of cells containing thousands of variants is possible in a single experiment, but even so, profiling variants using Sort-seq techniques remains costly, both in time and reagent/equipment expense. Yet the clear conceptual throughline from MAVE data generation to diagnostic yield primes these techniques to be substantially sharpened in the coming years.

Sortscore could be applied to score any continuous, sortable phenotype, so long as the technique doesn’t degrade the binned genetic material. This package could provide an easily accessible scoring framework for up and coming technologies that may expand what we refer to as Sort-seq. Sortable and multiplexable phenotypes might include additional spectroscopic, electrical, mechanical, or morphological cellular features. Responsibility lies with the user to determine if such a metric adequately captures the genotype-phenotype relationship.

### 4.3 Concluding remarks

We provide a clearly documented and accessible tool to serve as a starting point for analyzing Sort-seq counts data. Questions, bug reports, and feature requests may be directed to the project’s GitHub Issues page at https://github.com/dbaldridge-lab/sortscore. By releasing this package, we hope to lower the barrier to entry for groups exploring these experimental methods. We also hope to make validating the conclusions gleaned from Sort-seq equally approachable to groups that do not routinely use these methods, which may increase parity in the analysis protocols for Sort-seq variant effect assays compared to the more widely employed growth-based methods in use today.

## Supplementary Material

btag664_Supplementary_Data

## References

[btag664-B1] Aslanzadeh V , Brierley GV, Kumar R et al Deep mutational scanning of the human insulin receptor ectodomain to inform precision therapy for insulin resistance. Nat Commun. 2025;16:9143. 10.1038/s41467-025-64178-4 PMC12528385 41093840

[btag664-B2] Brnich SE , Rivera‐Muñoz EA, Berg JS. Quantifying the potential of functional evidence to reclassify variants of uncertain significance in the categorical and Bayesian interpretation frameworks. Human Mutat. 2018;39:1531–41. 10.1002/humu.23609 PMC6548460 30095857

[btag664-B3] Cock PJA , Antao T, Chang JT et al Biopython: freely available Python tools for computational molecular biology and bioinformatics. Bioinformatics (Oxford, England), 2009;25:1422–3. 10.1093/bioinformatics/btp163 PMC2682512 19304878

[btag664-B4] Çubuk H , Jin X, Phipson B et al Variant scoring tools for deep mutational scanning. Mol Syst Biol. 2025;21:1293–305. 10.1038/s44320-025-00137-x PMC12494760 40781375

[btag664-B5] Esposito D , Weile J, Shendure J et al MaveDB: an open-source platform to distribute and interpret data from multiplexed assays of variant effect. Genome Biol. 2019;20:223. 10.1186/s13059-019-1845-6 PMC6827219 31679514

[btag664-B6] Faure AJ , Lehner B. MoCHI: neural networks to fit interpretable models and quantify energies, energetic couplings, epistasis, and allostery from deep mutational scanning data. Genome Biol. 2024;25:303. 10.1186/s13059-024-03444-y PMC11610129 39617885

[btag664-B7] Faure AJ , Schmiedel JM, Baeza-Centurion P et al DiMSumml: an error model and pipeline for analyzing deep mutational scanning data and diagnosing common experimental pathologies. Genome Biol. 2020;21:207. 10.1186/s13059-020-02091-3 PMC7429474 32799905

[btag664-B8] Findlay GM , Daza RM, Martin B et al Accurate classification of BRCA1 variants with saturation genome editing. Nature. 2018;562:217–222. 10.1038/s41586-018-0461-z PMC6181777 30209399

[btag664-B9] Fowler DM , Fields S. Deep mutational scanning: a new style of protein science. Nat Methods. 2014;11:801–807. 10.1038/nmeth.3027 PMC4410700 25075907

[btag664-B10] Freudenberg J , Rao J, Howard MK et al Accurate variant effect estimation in FACS-based deep mutational scanning data with Lilace. Genome Biol. 2026;27:48. 10.1186/s13059-026-03934-1 PMC12918140 41593735

[btag664-B11] Gaber A , Lehner B, Baranowski C et al A strategy for scalable data collection of soluble protein expression in diverse hosts. *Zenodo*, 2024. https://zenodo.org/records/14014029

[btag664-B12] Gersing S , Schulze TK, Cagiada M et al Characterizing glucokinase variant mechanisms using a multiplexed abundance assay. Genome Biol. 2024;25:98. 10.1186/s13059-024-03238-2 PMC11021015 38627865

[btag664-B13] Gilliot P-A , Gorochowski TE. Effective design and inference for cell sorting and sequencing based massively parallel reporter assays. Bioinformatics. 2023;39:btad277. 10.1093/bioinformatics/btad277 PMC10182853 37084251

[btag664-B14] Harris CR , Millman KJ, van der Walt SJ et al Array programming with NumPy. Nature. 2020;585:357–62. 10.1038/s41586-020-2649-2 PMC7759461 32939066

[btag664-B15] Hunter JD. Matplotlib: a 2D graphics environment. Comput Sci Eng. 2007;9:90–5. 10.1109/MCSE.2007.55

[btag664-B16] Kinney JB. Using deep sequencing to characterize the biophysical mechanism of a transcriptional regulatory sequence. Proc Natl Acad Sci USA. 2010;107:9158–63. 10.1073/pnas.1004290107 PMC2889059 20439748

[btag664-B17] Lambrecht J , Cichocki N, Hübschmann T et al Flow cytometric quantification, sorting and sequencing of methanogenic archaea based on F420 autofluorescence. Microb Cell Fact. 2017;16:180. 10.1186/s12934-017-0793-7 PMC5663091 29084543

[btag664-B18] Livesey BJ , Marsh JA. Using deep mutational scanning to benchmark variant effect predictors and identify disease mutations. Mol Syst Biol. 2020;16:e9380. 10.15252/msb.20199380 PMC7336272 32627955

[btag664-B19] Ma K , Huang S, Ng KK et al Saturation mutagenesis-reinforced functional assays for disease-related genes. Cell. 2024;187:6707–24.e22. 10.1016/j.cell.2024.08.047 PMC11568926 39326416

[btag664-B20] Martin M. Cutadapt removes adapter sequences from high-throughput sequencing reads. EMBnet.Journal. 2011;17 :Article 1. 10.14806/ej.17.1.200

[btag664-B21] Matreyek KA , Starita LM, Stephany JJ et al Multiplex assessment of protein variant abundance by massively parallel sequencing. Nat Genet. 2018;50:874–82. 10.1038/s41588-018-0122-z PMC5980760 29785012

[btag664-B22] McEwen AE , Tejura M, Fayer S et al Multiplexed assays of variant effect for clinical variant interpretation. Nat Rev Genet. 2026;27:137–54. 10.1038/s41576-025-00870-x 40691352

[btag664-B23] Pavletich NP , Pabo CO. Zinc finger-DNA recognition: crystal structure of a Zif268-DNA complex at 2.1 A. Science. 1991;252:809–17. 10.1126/science.2028256 2028256

[btag664-B24] Peterman N , Levine E. Sort-seq under the hood: Implications of design choices on large-scale characterization of sequence-function relations. BMC Genomics. 2016;17:206. 10.1186/s12864-016-2533-5 PMC4784318 26956374

[btag664-B25] Popp NA , Powell RL, Wheelock MK et al Multiplex and multimodal mapping of variant effects in secreted proteins via MultiSTEP. Nat Struct Mol Biol. 2025;32:2099–111. 10.1038/s41594-025-01582-w PMC12373428 40514537

[btag664-B26] Pusapati GV , Kong JH, Patel BB et al CRISPR screens uncover genes that regulate target cell sensitivity to the morphogen sonic hedgehog. Dev Cell. 2018;44:113–29.e8. 10.1016/j.devcel.2017.12.003 PMC5792066 29290584

[btag664-B27] Rao J , Xin R, Macdonald C et al Rosace: a robust deep mutational scanning analysis framework employing position and mean-variance shrinkage. Genome Biol 2024;25:138. 10.1186/s13059-024-03279-7 PMC11127319 38789982

[btag664-B28] Rognes T , Flouri T, Nichols B et al VSEARCH: a versatile open source tool for metagenomics. PeerJ. 2016;4:e2584. 10.7717/peerj.2584 PMC5075697 27781170

[btag664-B29] Rubin AF , Gelman H, Lucas N et al A statistical framework for analyzing deep mutational scanning data. Genome Biol 2017;18:150. 10.1186/s13059-017-1272-5 PMC5547491 28784151

[btag664-B30] Shepherdson JL , Granas DM, Li J et al Mutational scanning of CRX classifies clinical variants and reveals biochemical properties of the transcriptional effector domain. Genome Res. 2024;34:1540–1552. 10.1101/gr.279415.124 PMC11529990 39322280

[btag664-B31] Tabet D , Parikh V, Mali P et al Scalable functional assays for the interpretation of human genetic variation. Annu Rev Genet. 2022;56:441–65. 10.1146/annurev-genet-072920-032107 36055970

[btag664-B32] Tareen A , Kooshkbaghi M, Posfai A et al MAVE-NN: learning genotype-phenotype maps from multiplex assays of variant effect. Genome Biol. 2022;23:98. 10.1186/s13059-022-02661-7 PMC9011994 35428271

[btag664-B33] Tejura M , Chen Y, McEwen AE et al A scalable approach to resolving variants of uncertain significance. bioRxiv. 2026. 10.64898/2026.02.14.705848

[btag664-B34] The pandas development team. pandas-dev/pandas: Pandas (Version v3.0.1) [Computer software]. *Zenodo*, 2026. 10.5281/ZENODO.3509134

[btag664-B35] Tonner PD , Pressman A, Ross D. Interpretable modeling of genotype–phenotype landscapes with state-of-the-art predictive power. Proc Natl Acad Sci. 2022;119:e2114021119. 10.1073/pnas.2114021119 PMC9245639 35733251

[btag664-B36] Virtanen P , Gommers R, Oliphant TE et al SciPy 1.0: fundamental algorithms for scientific computing in Python. Nat Methods. 2020;17:261–272. 10.1038/s41592-019-0686-2 PMC7056644 32015543

[btag664-B37] Waskom ML. Seaborn: statistical data visualization. J Open Source Softw. 2021;6:3021. 10.21105/joss.03021

[btag664-B38] Wehnert B , Stammnitz M. Nf-core/deepmutscan [Nextflow]. *nf-core*.2025. https://github.com/nf-core/deepmutscan (Original work published 2025).

[btag664-B39] Weile J , Ferra G, Boyle G et al Pacybara: accurate long-read sequencing for barcoded mutagenized allelic libraries. Bioinformatics. 2024;40:btae182. 10.1093/bioinformatics/btae182 PMC11021806 38569896

[btag664-B40] Weile J , Roth FP. Multiplexed assays of variant effects contribute to a growing genotype–phenotype atlas. Human Genet. 2018;137:665–678. 10.1007/s00439-018-1916-x PMC6153521 30073413

