# Supplementary material for: sortscore: Sort-seq MAVE scoring and visualization using Python
Source: Bioinformatics. 2026 Sep 2;42(10):btag664. doi: 10.1093/bioinformatics/btag664 (PMC13682839; doi:10.1093/bioinformatics/btag664)
Supplement: Supplementary file 1 [file btag664_supplementary_data.docx]

# Supplementary Table and Figures

| **Software** | **Assay /**  **Fitness Estimate** | **Method** | **Uncertainty Quantification** |
| --- | --- | --- | --- |
| FORECAST  (Gilliot & Gorochowski, 2023) | Sort-seq Massively Parallel Reporter Assay (MPRA); mean fluorescence from inferred distribution | Maximum likelihood estimation / method of moments | Confidence intervals from Fisher information, delta method |
| Enrich2  (Rubin et al., 2017) | Time-course growth or selection assay; log-enrichment ratio or slope from weighted least-squares regression | Mixed effects model | Random effects term |
| DiMSum  (Faure et al., 2020) | Growth DMS  (two-population);  WT-normalized log-enrichment ratio | Replicate enrichment with interpretable error model | Error model with additive and multiplicative terms |
| Rosace  (Rao et al., 2024) | Growth DMS (two-population or time-series); slope from Bayesian hierarchical regression | Bayesian shrinkage | Posterior intervals |
| TileSeqMave  (Gersing et al., 2024; Weile & Roth, 2018) | Tiled selection DMS; bias-corrected log-enrichment score | Error-weighted averaging | Bayesian refinement or regularized standard error |

**Supplementary Table 1:** Comparison of a selection of MAVE analytical tools that provide variant-level activity scoring, including software tailored to process growth-based assays.

**Supplementary Figure 1:** Overview of Sort-seq experimental workflow and analysis. Created in BioRender. Baldridge, D. (2026) [https://BioRender.com/a6qlfju](https://biorender.com/a6qlfju)

**A**

**B**

**Supplementary Figure 2:** (A) Schematic of sequential gating strategy used for flow cytometry analysis. Sequential gating was performed to identify live single cells by first gating on the FSC Area-SSC Area population, followed by singlet discrimination using FSC Area-FSC Width and SSC Area-SSC Width. (B) The mixed distribution of fluorescing cells containing the DMS library is gated for sorting. Median fluorescence intensity (M_i_) and variant counts in each bin (B_i_) are used by sortscore to estimate mean variant activity.

**Supplementary Figure 3:** Comparison of MFI and ordinal bin-weighted sortscore estimates. Variant scores from a FACS-based deep mutational scan of GLI2 residues 551–583 were calculated using either median fluorescence intensity (MFI) or ordinal values as bin weights. Each point represents an amino acid variant (A: Spearman’s ρ = 0.93; n = 695) or DNA variant (B: ρ = 0.98; n = 2081).

**Supplementary Figure 4:** There are three normalization options available in sortscore, named as “linear range normalization” (as used in the BRCA1 DMS), “two-pole,” and “one-pole.” The default two-pole normalization method is demonstrated here. (A) Heatmaps of GLI2 DNA Binding Domain DMS raw scores from two tiles of a tiled experiment. Prior to normalization, batch effects are apparent across the separate Sort-seq experiments. (B) “Two-pole” normalization option anchors scores on positive and negative controls and reduces apparent batch effects across tiles.

**Supplementary Figure 5:** Comparison of sortscore-derived scores (using default sortscore options) to reported scores for the CRX transcription factor DMS dataset (Shepherdson et al., 2024). **A:** Scatterplot of normalized reported activity scores versus sortscore mean fluorescence scores (n = 5,374; Spearman's ρ = 0.938). **B:** Heatmap showing sortscore-derived results for the CRX transcription factor.

**Supplementary Figure 6:** Comparison of sortscore and Lilace variant-effect estimates. Variant scores from a FACS-based deep mutational scan of GLI2 residues 518–550 were analyzed using sortscore and Lilace. Each point represents one variant and is colored by mutation type. Estimates from the two methods were strongly correlated (Spearman’s ρ = 0.99; n = 693 variants).
